# Tumor antigens and immune subtypes guided mRNA vaccine development for kidney renal clear cell carcinoma

**DOI:** 10.1186/s12943-021-01465-w

**Published:** 2021-12-06

**Authors:** Hang Xu, Xiaonan Zheng, Shiyu Zhang, Xianyanling Yi, Tianyi Zhang, Qiang Wei, Hong Li, Jianzhong Ai

**Affiliations:** 1 Institute of Urology, West China Hospital, Sichuan University, Chengdu, 610041 China; 2 Department of Urology, West China Hospital, Sichuan University, Chengdu, 610041 China; 3 Institute of Systems Genetics, West China Hospital, Sichuan University, Chengdu, 610041 China

**Keywords:** mRNA vaccine, Kidney renal clear cell carcinoma, Immunotherapy, Tumor antigens, Immune subtypes, Immune landscape

## Abstract

Current treatment strategy for kidney renal clear cell carcinoma (KIRC) is limited. Tumor-associated antigens, especially neoantigen-based personalized mRNA vaccines represent new strategies and manifest clinical benefits in solid tumors, but only a small proportion of patients could benefit from them, which prompts us to identify effective antigens and suitable populations to facilitate mRNA vaccines application in cancer therapy. Through performing expression, mutation, survival and correlation analyses in TCGA-KIRC dataset, we identified four genes including DNA topoisomerase II alpha (TOP2A), neutrophil cytosol factor 4 (NCF4), formin-like protein 1 (FMNL1) and docking protein 3 (DOK3) as potential KIRC-specific neoantigen candidates. These four genes were upregulated, mutated and positively associated with survival and antigen-presenting cells in TCGA-KIRC. Furthermore, we identified two immune subtypes, named renal cell carcinoma immune subtype 1 (RIS1) and RIS2, of KIRC. Distinct clinical, molecular and immune-related signatures were observed between RIS1 and RIS2. Patients of RIS2 had better survival outcomes than those of RIS1. Further comprehensive immune-related analyses indicated that RIS1 is immunologically “hot” and represent an immunosuppressive phenotype, whereas RIS2 represents an immunologically “cold” phenotype. RIS1 and RIS2 also showed differential features with regard to tumor infiltrating immune cells and immune checkpoint-related genes. Moreover, the immune landscape construction identified the immune cell components of each KIRC patient, predicted their survival outcomes, and assisted the development of personalized mRNA vaccines. In summary, our study identified TOP2A, NCF4, FMNL1 and DOK3 as potential effective neoantigens for KIRC mRNA vaccine development, and patients with RIS2 tumor might benefit more from mRNA vaccination.

**Supplementary Information:**

The online version contains supplementary material available at 10.1186/s12943-021-01465-w.

## Background

Kidney renal clear cell carcinoma (KIRC) occupies about 85% of renal cell carcinoma (RCC) [1]. The prognosis of KIRC, compared to other histological subtypes, was generally worse before adjustment of tumor stage and grade [2, 3]. Therefore, the need of new strategies treating KIRC has become necessary and urgent. The use of mRNA-based vaccine has been proposed as a promising approach of combatting tumors two decades ago, and has again become a hotspot under the background of coronavirus disease-2019 (COVID-19) pandemic [4–7]. However, the application of mRNA vaccine in KIRC is somehow lagged. Although a certain degree of immune response was observed, only a limited number of studies have investigated mRNA vaccine in KIRC [8, 9] and their results were still far from satisfactory. Hence, the present study aims to explore novel candidate tumor antigens for KIRC mRNA vaccine development. Additionally, immune subtypes of KIRC will be classified and patients suitable for mRNA vaccination will be identified. Taken together, this study may pave an avenue for the development of KIRC mRNA vaccine and the identification of KIRC patients suitable for mRNA vaccination.

## Results and discussion

### Screening of candidate antigens in KIRC

**Fig. 1:**
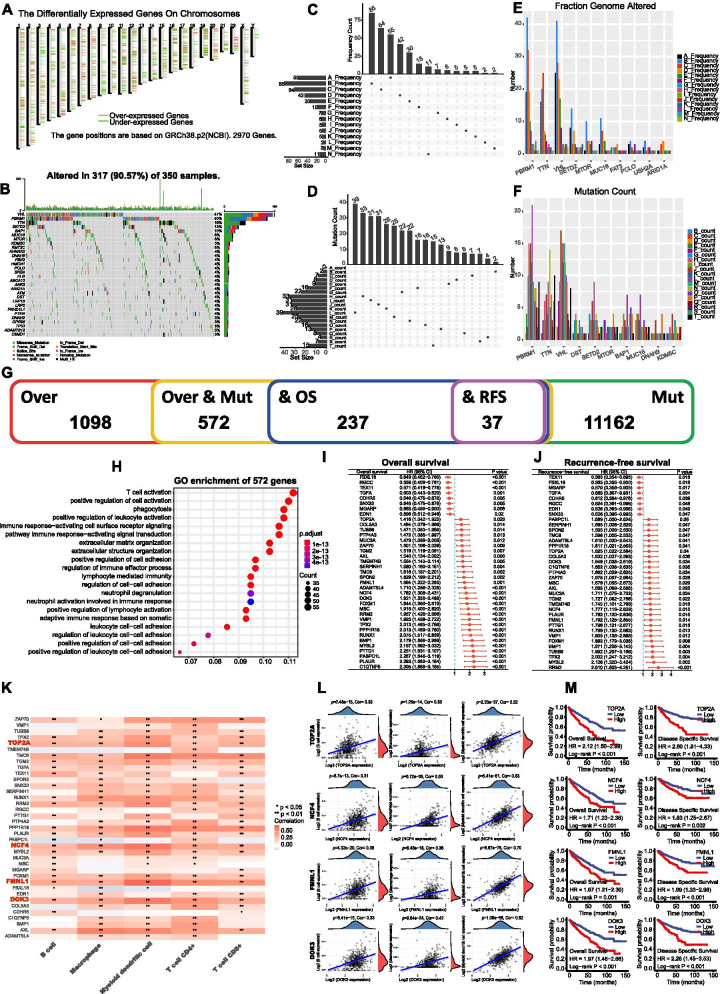
Identification of potential antigens in KIRC. **a** Chromosomal distribution of up- and down-regulated genes in KIRC; **b** Waterfall diagram of the top 30 mutant genes; **c** Distribution of mutation frequency; **d** Distribution of mutation number; **e** Distribution of mutation number of the top 10 genes; **f** Distribution of mutation frequency of the top 10 genes. **g** Overlapped genes identified through intersection; **h** GO enrichment analysis of 572 genes after intersection of overexpressed and mutated genes; **i-j** Univariate Cox regression analysis of the 37 potential antigens for OS (**i**) and RFS (**j**). **k** Correlation analysis of 37 genes with immune infiltrating cells, red box indicates genes closely related to APCs (threshold: spearman correlation coefficient > 0.3); **l** Association of TOP2A, MCF4, FMNL1 and DOK3 with B cell, macrophage, and dendritic cells; **m** Kaplan-Meier curves of the association of TOP2A, MCF4, FMNL1 and DOK3 with OS and DSS. GO, Gene Ontology; KEGG, Kyoto Encyclopedia of Genes and Genomes; OS, overall survival; RFS, recurrence-free survival; DSS, disease specific survival. * *p* < 0.05 and ** *p* < 0.01

The workflow of our study was presented in Fig. S1. A total of 1098 aberrantly expressed genes were identified (Fig. S2a-b) and the chromosomes distribution of these genes were showed in Fig. 1a. Next, we identified 11,162 genes that mutated in KIRC (Fig. S2c) and the top 30 mutated genes were showed in Fig. 1b. The altered genome fraction and mutation counts in individual samples were demonstrated in Fig. 1c-d. Of note, PBRM1, TTN and VHL were also the most frequently mutated genes considering both altered genome fraction and mutation counts (Fig. 1e-f). Combining the expression and mutation data of KIRC, 572 genes that were highly expressed and mutated in KIRC were identified as potential candidate antigens (Fig. 1g). The Gene Ontology (GO) analysis demonstrated that these 572 genes involved in immune response-related pathways (Fig. 1h**,** Fig. S2d-f). The results indicated that these genes were aberrantly highly expressed and mutated in KIRC, which might stimulate tumor-specific immune response. Thus, the 572 genes were the potential candidates for mRNA vaccine development.

To explore the key genes that functioned as best candidates for mRNA vaccine targets, we further identified 37 genes which were associated both OS and RFS from the 572 genes (Fig. 1i-j). Given the pivotal role of antigen-presenting cells (APCs) in the function of mRNA vaccines, we analyzed the association of these 37 genes with APCs using single sample gene set enrichment analysis (ssGSEA) [10] (Fig. 1k). Finally, 4 genes including TOP2A, FCN4, FMNL1 and DOK3 that were closely associated with APCs were identified (spearman correlation coefficient > 0.3; Fig. 1l). All of these four genes were positively associated with APCs and could serve as potential tumor antigens that can be recognized and processed by the APCs to T cells, finally triggering strong immune response against tumor cells. More importantly, the survival analysis demonstrated that the high expression of these four genes were associated with decreased survival in KIRC (Fig. 1m**),** suggesting the four genes were of importance in KIRC development and progression. Taken together, TOP2A, NCF4, FMNL1 and DOK3 were significantly upregulated, mutated and positively associated with APCs infiltration in KIRC. Therefore, mRNA vaccines encoding these 4 genes might induce anti-tumor immune response and eliminate malignant cells.

### Immune subtypes identification

**Fig. 2:**
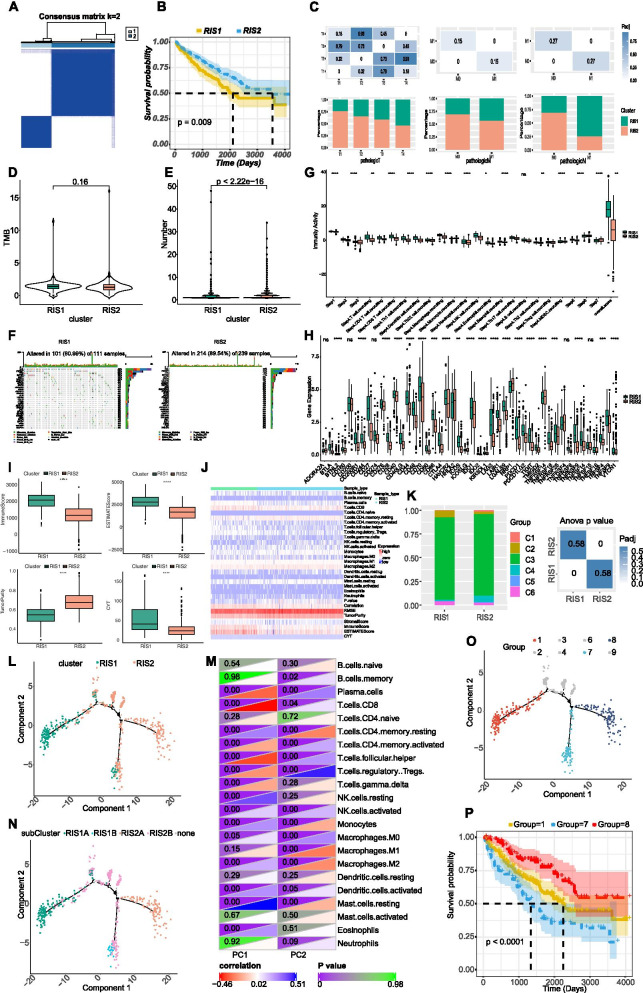
Identification of immune subtypes in KIRC. **a** Sample clustering heatmap; **b** Kaplan-Meier curves of the association of immune subtypes with overall survival; **c** Association of immune subtype with pathological T, N, and M stage. RIS, renal cancer immune subtype; **d-e** Association of immune subtypes with TMB (d) and mutation number (e); **f** Top 30 mutated genes in RIS1 and RIS2; **g** Distribution of immune activity scores in RIS1 and RIS2; **h** Expression of immune checkpoints between RIS1 and RIS2; **i** Association of immune subtypes with immune score, estimate score, tumor purity, and CYT; **j** Immune infiltration score heatmap; **k** Relationship between immune subtypes and existing pan-cancer immune subtypes; **l** Immune landscape in KIRC. Each dot represents one patient, and the immune subtype is color coded. The horizontal axis represents the first principal component, and the vertical axis represents the second principal component; **m** The heatmap of the correlation between the two principal components and immune cells; **n** The immune landscape of subgroup of KIRC immune subtype; **o** The immune landscape of samples from three extreme positions; **p** The prognosis of three extreme positions. CYT, immune cytolytic activity; RIS, renal cancer immune subtype; ns, not significant. * *p* < 0.05, ** *p* < 0.01, *** *p* < 0.001 and **** *p* < 0.0001

Tumor immune microenvironment might impact the efficacy of immunotherapy and immune subtypes might be helpful to identify patients who can response to mRNA vaccination. We analyzed the expression profiles of 1621 immune-related genes in KIRC samples to construct consistent clusters. We selected k = 2 (Fig. S3a-b) for stable clustering of immune-related genes and obtained two immune subtypes, named kidney renal cell carcinoma immune subtype 1 (RIS1) and RIS2, respectively (Fig. 2a). The principal component analysis (PCA) validated that these two subtypes could be well distinguished (Fig. S3c). Survival analysis revealed that RIS1 had significant worse prognosis than RIS2 (Fig. 2b) and RIS1 had significant higher pathological T, N and M stages than RIS2 (Fig. 2c). Overall, our immune subtyping was well distinguished and could be applied to identify KIRC patients with better pathological and survival outcomes.

### Immune subtypes with tumor mutation burden (TMB)

As TMB was associated with immunotherapy response [11], we assessed the TMB, mutation counts and copy number alteration (CNA) status between RIS1 and RIS2. Our results showed that there was no difference between groups regarding TMB (Fig. 2d), while RIS1 had significantly higher mutation number than RIS2 (Fig. 2e). The waterfall diagrams of different subtypes were showed in Fig. 2f. The CNA was found to be significantly higher in RIS1 than in RIS2 (Fig. S3d-f). These findings suggested that RIS2 might be more likely to respond for mRNA vaccines. Furthermore, we found that RIS2 had significantly lower immunity activity than RIS1 (Fig. 2g), suggesting the mRNA vaccine targeting RIS2 might reinforce its immune response.

### Immune subtypes with immune checkpoints (ICPs) and immune infiltrating cells

Given the pivotal role of ICPs in immune response regulation, which might impact the anti-tumor efficacy of mRNA vaccines, we analyzed the expression of ICPs between two subtypes (Fig. 2h). Our results demonstrated that 35 (77.8%) of ICPs, such as CD274, CTLA4, PDCD1, IDO1 and TIGIT, were significantly higher in RIS1 than RIS2, suggesting mRNA vaccines might function better in RIS2 for its relatively low ICPs expressions. Moreover, we showed that RIS1 had markedly higher immune score, higher stromal score and lower tumor purity (Fig. 2i) than RIS2. In addition, we also conducted cytolytic activity (CYT) analysis and the results showed that RIS1 had higher CYT than RIS2 (Fig. 2i). Furthermore, we analyzed the differences of 28 immune infiltrating cells between groups (Fig. 2j). The results showed that plasma cells, CD8 T cells, memory CD4 T cells were markedly higher in RIS1 than RIS2 (Fig. S4). Therefore, RIS1 is immunologically “hot” and represent an immunosuppressive phenotype, whereas RIS2 represents an immunologically “cold” phenotype. Previous study [12] had identified six pan-cancer subtypes, including C1 (Wound Healing), C2 (IFN-γ Dominant), C3 (Inflammatory), C4 (Lymphocyte Depleted), C5 (Immunologically Quiet) and C6 (TGF-β Dominant). The distributions of these six types in RIS1 and RIS2 were analyzed as well. As exhibited in Fig. 2k**,** RIS1 had significantly higher proportion of C2 than RIS2, while RIS2 had significantly higher proportion of C4 than RIS1. This result further validated the “hot” phenotype of RIS1 and the “cold” phenotype of RIS2. Therefore, mRNA vaccine administration in RIS2 might stimulate the immune response, namely turning “cold” tumor to “hot”.

### Immune landscape of KIRC

The expression profile of immune-related genes in each KIRC sample was selected to construct the immune landscape of KIRC. We found that the point distribution in RIS1 and RIS2 is relatively discrete (Fig. 2l). Principal component 1 (PC1, horizontal axis) was most negatively correlated with plasma cells, CD8 T cells and T follicular helper cells, and most positively correlated with resting NK cells and resting mast cells. On the contrary, principal component 2 (PC2, vertical axis) was most negatively correlated with resting memory T cells and most positively correlated with Tregs (Fig. 2m). The correlation of different immune cells between PC1 and PC2 further indicated the accuracy of our classification. Moreover, heterogeneities in each cluster can be observed from Fig. 2l that even RIS1 and RIS2 also exhibited opposing distribution. Therefore, we further divided RIS1 and RIS2 into two subgroups according to the distribution location of immune cell groups (Fig. 2n). RIS1B and RIS2A had significantly lower CD8 T cells compared with their counterparts, suggesting mRNA vaccines might be viable in RIS1B and RIS2A (Fig. S5a-b). In addition, by comparing the prognosis of samples with extreme distribution in the immune landscape, we found that patients in group 8 had best survival outcomes and patients in group 7 had worst survival outcomes (Fig. 2o-p). Taken together, construction of immune landscape of KIRC enabled us to accurately identify the immune cell components of each KIRC patients and predict their survival outcomes, finally assist the development of personalized mRNA vaccines.

### Identification of immune gene co-expression modules and immune hub genes of KIRC

The immune gene co-expression module clustered the KIRC samples through weighted gene coexpression network analysis (WGCNA, Fig. S6a-d). We subsequently analyzed the distribution of characteristic genes of the RIS1 and RIS2 in these 9 modules (Fig. S6e). RIS1 showed the higher eigengenes in black, brown, magenta, pink, red and turquoise modules than RIS2. Survival analysis showed that blue, yellow and green modules were associated with overall survival (OS) in KIRC (Fig. S7a). KEGG analysis showed that T cell receptor signaling pathway was enriched in blue module, cytokine and cytokine receptor interaction in yellow and green module (Fig. S7b-d). The blue module was picked up to build the risk score. Firstly, we selected the hub genes including RDX、IREB2、UBR1 and PIK3CA from the module (MM > 0.9). We found that these four hub genes were all mutated in KIRC. A risk model was also built (Fig. S8a-b). It was found that the risk score had significant prognostic efficacy (Fig. S8c). The expression of these four genes was visualized by heatmap (Fig. S8d). Thus, the hub genes identified in this study can serve as the prognostic factors for KIRC and serve as biomarkers for picking up KIRC patients for mRNA vaccines.

### Identification of differential expression genes (DEGs) between immune subtypes

To explore our immunotyping more deeply, 107 genes were identified as the DEGs between RIS1 and RIS2 (Fig. S9a). Pathway enrichment analyses were showed in Fig. S9b-e and they were enriched in immune response pathways such as “active immune response”, “lymphocyte mediated immunity” and “humoral immune response”, further indicating that our immunotyping could reflect the antitumor immune response in KIRC. We next performed univariate Cox regression analysis on these 107 DEGs and 31 genes were significantly correlated with decreased survival outcomes (threshold *P* < 0.01, Fig. S10a). Then we performed lasso regression analysis on these 31 genes and finally obtained 7 high-risk genes (CCL19, CCL5, IGLV9–19, IGLV3–27, IGLV3–21, IGLC2 and IGHG3, Fig. S10b-d). Of note, two of these 7 genes (IGHG3 and IGIC2) were highly expressed and mutated genes in KIRC. A risk model was built based on the 7 genes according to the median of risk score (Fig. S10e-f) and the survival curves indicated the risk model had prognostic efficacy (Fig. S10g). The expression heatmap of these 7 genes was showed in Fig. S10h. It can be found that RIS1 had significantly higher scores and greater percentage of high-risk samples than RIS2 (Fig. S10i-j). The expression of the above 7 genes in TCGA-SKCM were picked up and calculated the risk score of each sample to build a model. The results showed that high risk group had significantly higher anti-CTLA-4 and anti-PD-1 response than low risk group (Fig. S10k), indicating that RIS2 had lower immune checkpoint efficacy than RIS1. Thus, immune checkpoint blockade might not suitable for KIRC patients with RIS2 and mRNA vaccines might be effective for RIS2 populations.

## Conclusions

In summary, our study identified TOP2A, NCF4, FMNL1 and DOK3 as potential effective neoantigens for KIRC mRNA vaccine development, and patients with RIS2 tumor might benefit more from mRNA vaccination. Our study paved a way for future mRNA vaccine development and define the suitable population for vaccination.

### Methods and availability of supporting data

Methods and materials used in our study are attached as supplementary information. All data are freely available from the public databases and the other necessary and reasonable information could be obtained from the corresponding author.

## Supplementary Information

**Additional file 1: Figure S1.** The workflow of the study. OE, overexpressed genes; APCs, antigen-presenting cells; TMB, tumor mutation burden; CNV, copy number alterations; DEGs, differentially expressed genes; RIS, renal cancer immune subtype. **Figure S2. a,** volcano plot; **b**, heatmap of overexpressed genes in normal and KIRC samples; **c,** overlapped genes identified through intersection; **d-f**, KEGG (**d**), Hallmark (**e**) and reactome (**f**) enrichment analysis of 572 genes after intersection of overexpressed and mutated genes. KEGG, Kyoto Encyclopedia of Genes and Genomes. **Figure S3. a**, cumulative distribution function curve; **b**, delta area of immune-related genes; **c**, principal component analysis; **d**, association of immune subtypes with G-score; **e-f**, Bar graph of copy number variation in RIS1 (**e**) and RIS2 (**f**). **Figure S4.** the differences of immune infiltration score among subtypes in immune cells. **Figure S5. a-b**, the differential enrichment fraction of immune cells in the above subgroups. RIS, renal cancer immune subtype; ns, not significant. * *p* < 0.05, ** *p* < 0.01, *** *p* < 0.001 and **** *p* < 0.0001. **Figure S6.** WGCNA module identification. **a**, sample clustering; **b**, scale-free fitting index of various soft threshold powers (β); **c**, the average connectivity; **d**, Dendrogram of all differentially expressed genes clustered based on a dissimilarity measure (1-TOM). **e**, number of genes in each module; **f**, difference distribution of feature vectors of each module in RIS1 and RIS2. RIS, renal cancer immune subtype; ns, not significant. * *p* < 0.05, ** *p* < 0.01, *** *p* < 0.001 and **** *p* < 0.0001. **Figure S7.** Identification of immune hub genes in KIRC. **a**, univariate Cox regression analysis of the 10 modules; **b-d**, Gene Ontology analysis of Blue (**b**), Yellow (**c**) and Green (**d**). **Figure S8. a**, risk score distribution; **b**, survival state distribution; **c,** prognosis of risk models; **d**, heatmap of RDX, IREB2, UBR1 and PIK3CA. **Figure S9. a,** heatmap of differentially expressed genes in immune subtypes; **b-e**, GO (**b-d**) and KEGG (**e**) enrichment of differentially expressed genes in immune subtypes. GO, gene ontology; KEGG, Kyoto Encyclopedia of Genes and Genomes. **Figure S10. a**, univariate Cox regression analysis; **b**-**d**, Lasso regression model; **e**, risk score distribution; **f**, survival state distribution; **g**, Survival curves of risk model; **h**, heatmap of genes in the risk model; **i**, risk score distribution between immune subtypes; **j**, Distribution of high and low risk group samples among subtypes; **k**, immune response inference based on SKCM. RIS, renal cancer immune subtype; SKCM, human skin cutaneous melanoma; CTLA-4, Cytotoxic T-Lymphocyte Associated Protein 4; PD-1, programmed cell death protein 1; nonR, non-responder; R, responder.

## Abbreviations

ccRCC: Clear cell renal cell carcinoma
KIRC: Kidney renal clear cell carcinoma
mRNA: Messenger ribonucleic acid
TCGA: The Cancer Genome Atlas
GO: Gene ontology
KEGG: Kyoto encyclopedia of genes and genomes
PCA: Principal component analysis
ssGSEA: Single sample gene set enrichment analysis
CYT: Cytolytic activity
RIS: KIRC immune subtype
CNA: Copy number alteration
TMB: Tumor mutation burden
ICP: Immune-checkpoint
WGCNA: Weighted gene coexpression network analysis
DEG: Differential expression gene
PFS: Progression-free survival
OS: Overall survival
APCs: Antigen-presenting cells
ICB: Immune checkpoint blockade
SKCM: Skin cutaneous melanoma
