# Supplementary figures and images for: Tumor antigens and immune subtypes guided mRNA vaccine development for kidney renal clear cell carcinoma
Source: Mol Cancer. 2021 Dec 6;20:159. doi: 10.1186/s12943-021-01465-w (PMC8645676; doi:10.1186/s12943-021-01465-w)

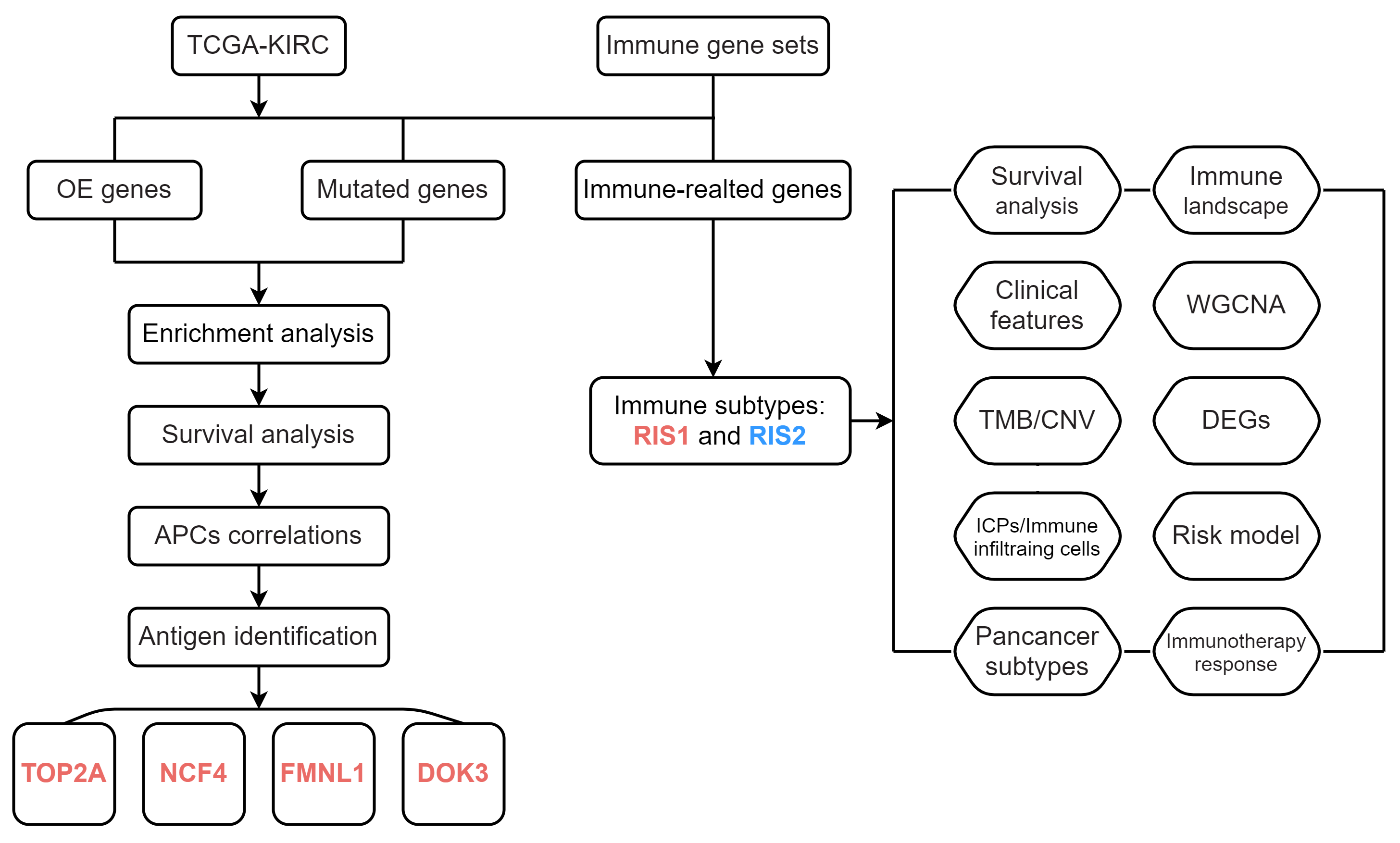

Supplement: Supplementary file 1 — Additional file 1: Figure S1. The workflow of the study. OE, overexpressed genes; APCs, antigen-presenting cells; TMB, tumor mutation burden; CNV, copy number alterations; DEGs, differentially expressed genes; RIS, renal cancer immune subtype. Figure S2. a, volcano plot; b, heatmap of overexpressed genes in normal and KIRC samples; c, overlapped genes identified through intersection; d-f, KEGG (d), Hallmark (e) and reactome (f) enrichment analysis of 572 genes after intersection of overexpressed and mutated genes. KEGG, Kyoto Encyclopedia of Genes and Genomes. Figure S3. a, cumulative distribution function curve; b, delta area of immune-related genes; c, principal component analysis; d, association of immune subtypes with G-score; e-f, Bar graph of copy number variation in RIS1 (e) and RIS2 (f). Figure S4. the differences of immune infiltration score among subtypes in immune cells. Figure S5. a-b, the differential enrichment fraction of immune cells in the above subgroups. RIS, renal cancer immune subtype; ns, not significant. * p < 0.05, ** p < 0.01, *** p < 0.001 and **** p < 0.0001. Figure S6. WGCNA module identification. a, sample clustering; b, scale-free fitting index of various soft threshold powers (β); c, the average connectivity; d, Dendrogram of all differentially expressed genes clustered based on a dissimilarity measure (1-TOM). e, number of genes in each module; f, difference distribution of feature vectors of each module in RIS1 and RIS2. RIS, renal cancer immune subtype; ns, not significant. * p < 0.05, ** p < 0.01, *** p < 0.001 and **** p < 0.0001. Figure S7. Identification of immune hub genes in KIRC. a, univariate Cox regression analysis of the 10 modules; b-d, Gene Ontology analysis of Blue (b), Yellow (c) and Green (d). Figure S8. a, risk score distribution; b, survival state distribution; c, prognosis of risk models; d, heatmap of RDX, IREB2, UBR1 and PIK3CA. Figure S9. a, heatmap of differentially expressed genes in immune subtypes; b-e, GO ( [file 12943_2021_1465_MOESM1_ESM.zip › Figure S1.tif]

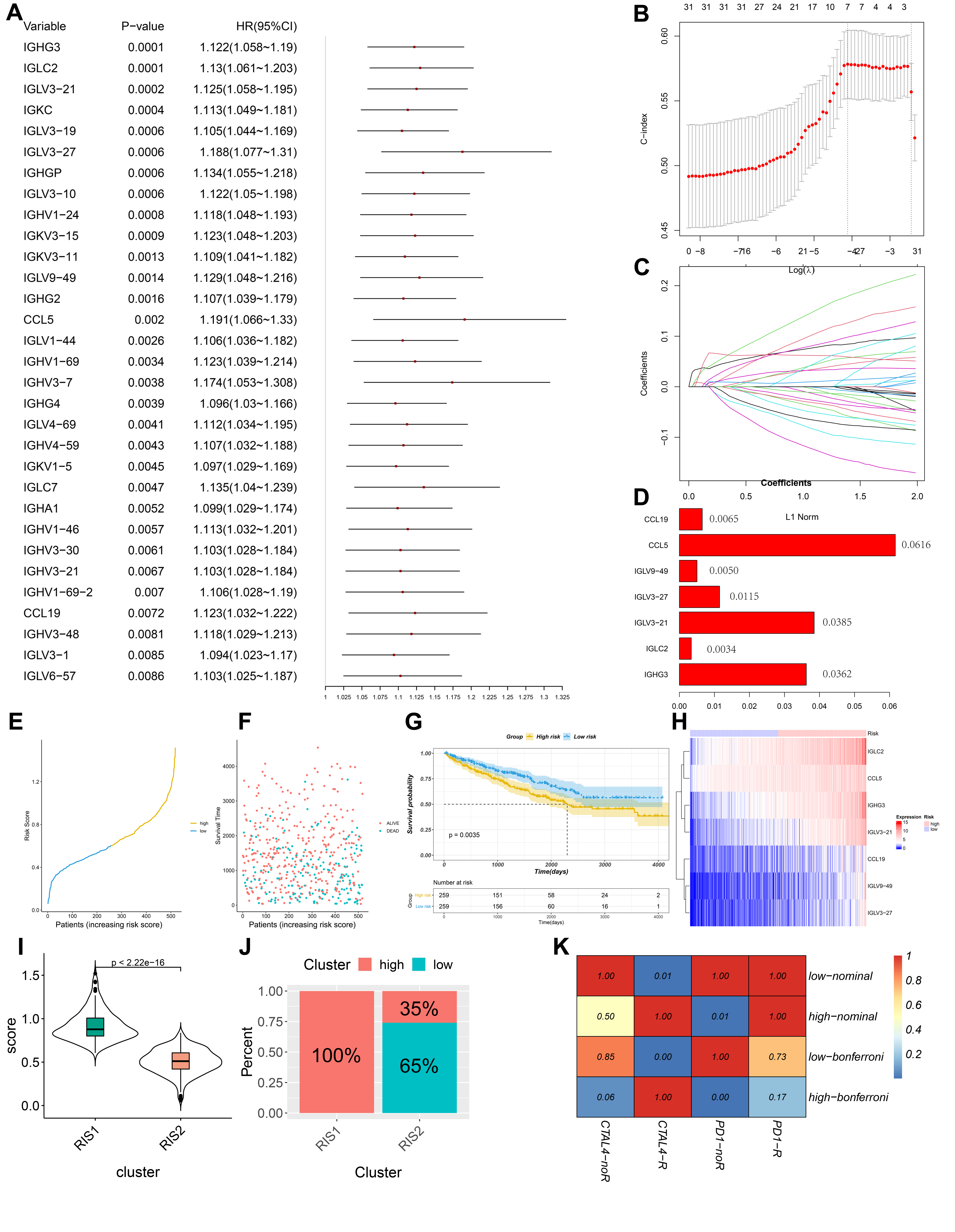

Supplement: Supplementary file 1 — Additional file 1: Figure S1. The workflow of the study. OE, overexpressed genes; APCs, antigen-presenting cells; TMB, tumor mutation burden; CNV, copy number alterations; DEGs, differentially expressed genes; RIS, renal cancer immune subtype. Figure S2. a, volcano plot; b, heatmap of overexpressed genes in normal and KIRC samples; c, overlapped genes identified through intersection; d-f, KEGG (d), Hallmark (e) and reactome (f) enrichment analysis of 572 genes after intersection of overexpressed and mutated genes. KEGG, Kyoto Encyclopedia of Genes and Genomes. Figure S3. a, cumulative distribution function curve; b, delta area of immune-related genes; c, principal component analysis; d, association of immune subtypes with G-score; e-f, Bar graph of copy number variation in RIS1 (e) and RIS2 (f). Figure S4. the differences of immune infiltration score among subtypes in immune cells. Figure S5. a-b, the differential enrichment fraction of immune cells in the above subgroups. RIS, renal cancer immune subtype; ns, not significant. * p < 0.05, ** p < 0.01, *** p < 0.001 and **** p < 0.0001. Figure S6. WGCNA module identification. a, sample clustering; b, scale-free fitting index of various soft threshold powers (β); c, the average connectivity; d, Dendrogram of all differentially expressed genes clustered based on a dissimilarity measure (1-TOM). e, number of genes in each module; f, difference distribution of feature vectors of each module in RIS1 and RIS2. RIS, renal cancer immune subtype; ns, not significant. * p < 0.05, ** p < 0.01, *** p < 0.001 and **** p < 0.0001. Figure S7. Identification of immune hub genes in KIRC. a, univariate Cox regression analysis of the 10 modules; b-d, Gene Ontology analysis of Blue (b), Yellow (c) and Green (d). Figure S8. a, risk score distribution; b, survival state distribution; c, prognosis of risk models; d, heatmap of RDX, IREB2, UBR1 and PIK3CA. Figure S9. a, heatmap of differentially expressed genes in immune subtypes; b-e, GO ( [file 12943_2021_1465_MOESM1_ESM.zip › Figure S10.tif]

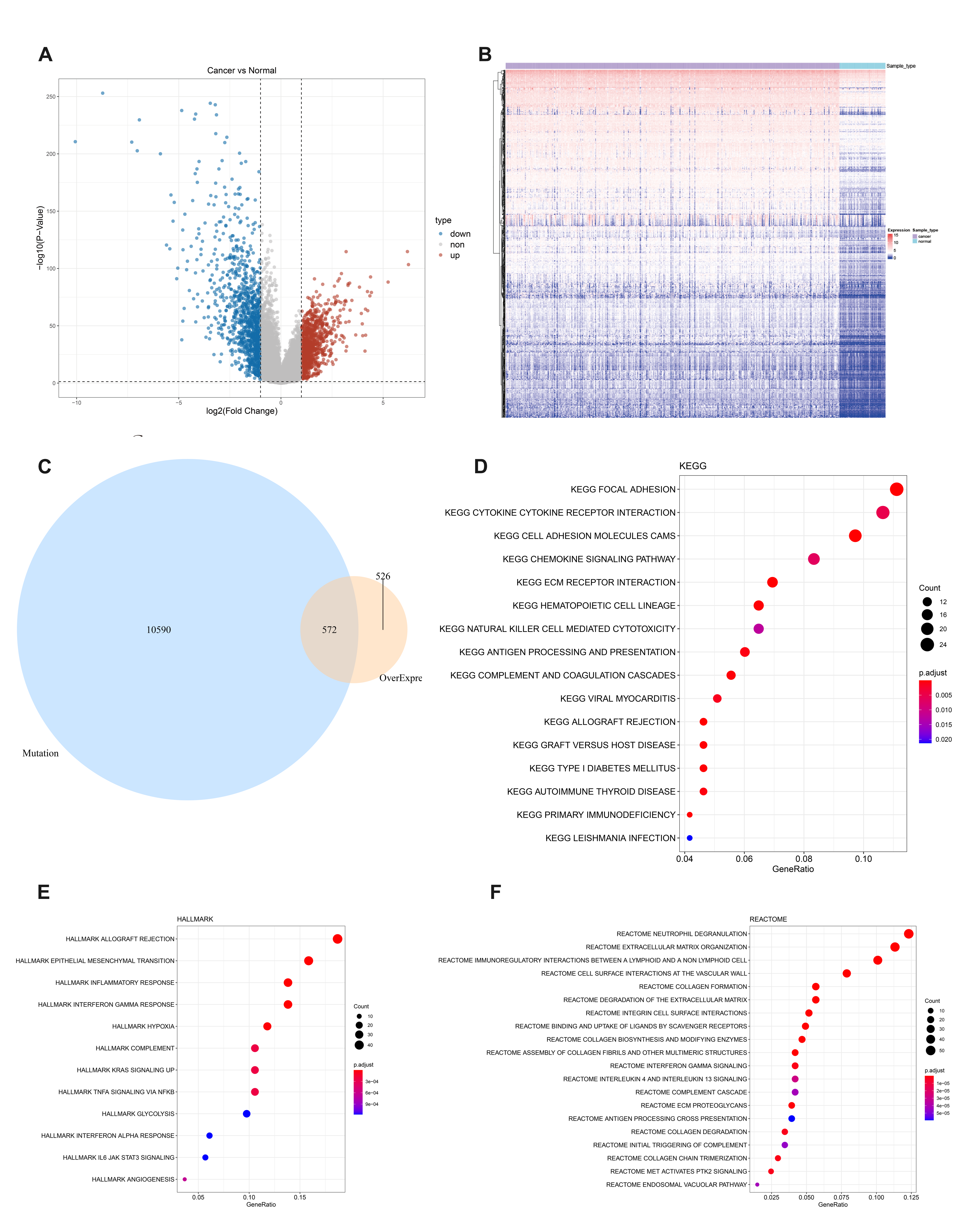

Supplement: Supplementary file 1 — Additional file 1: Figure S1. The workflow of the study. OE, overexpressed genes; APCs, antigen-presenting cells; TMB, tumor mutation burden; CNV, copy number alterations; DEGs, differentially expressed genes; RIS, renal cancer immune subtype. Figure S2. a, volcano plot; b, heatmap of overexpressed genes in normal and KIRC samples; c, overlapped genes identified through intersection; d-f, KEGG (d), Hallmark (e) and reactome (f) enrichment analysis of 572 genes after intersection of overexpressed and mutated genes. KEGG, Kyoto Encyclopedia of Genes and Genomes. Figure S3. a, cumulative distribution function curve; b, delta area of immune-related genes; c, principal component analysis; d, association of immune subtypes with G-score; e-f, Bar graph of copy number variation in RIS1 (e) and RIS2 (f). Figure S4. the differences of immune infiltration score among subtypes in immune cells. Figure S5. a-b, the differential enrichment fraction of immune cells in the above subgroups. RIS, renal cancer immune subtype; ns, not significant. * p < 0.05, ** p < 0.01, *** p < 0.001 and **** p < 0.0001. Figure S6. WGCNA module identification. a, sample clustering; b, scale-free fitting index of various soft threshold powers (β); c, the average connectivity; d, Dendrogram of all differentially expressed genes clustered based on a dissimilarity measure (1-TOM). e, number of genes in each module; f, difference distribution of feature vectors of each module in RIS1 and RIS2. RIS, renal cancer immune subtype; ns, not significant. * p < 0.05, ** p < 0.01, *** p < 0.001 and **** p < 0.0001. Figure S7. Identification of immune hub genes in KIRC. a, univariate Cox regression analysis of the 10 modules; b-d, Gene Ontology analysis of Blue (b), Yellow (c) and Green (d). Figure S8. a, risk score distribution; b, survival state distribution; c, prognosis of risk models; d, heatmap of RDX, IREB2, UBR1 and PIK3CA. Figure S9. a, heatmap of differentially expressed genes in immune subtypes; b-e, GO ( [file 12943_2021_1465_MOESM1_ESM.zip › Figure S2.tif]

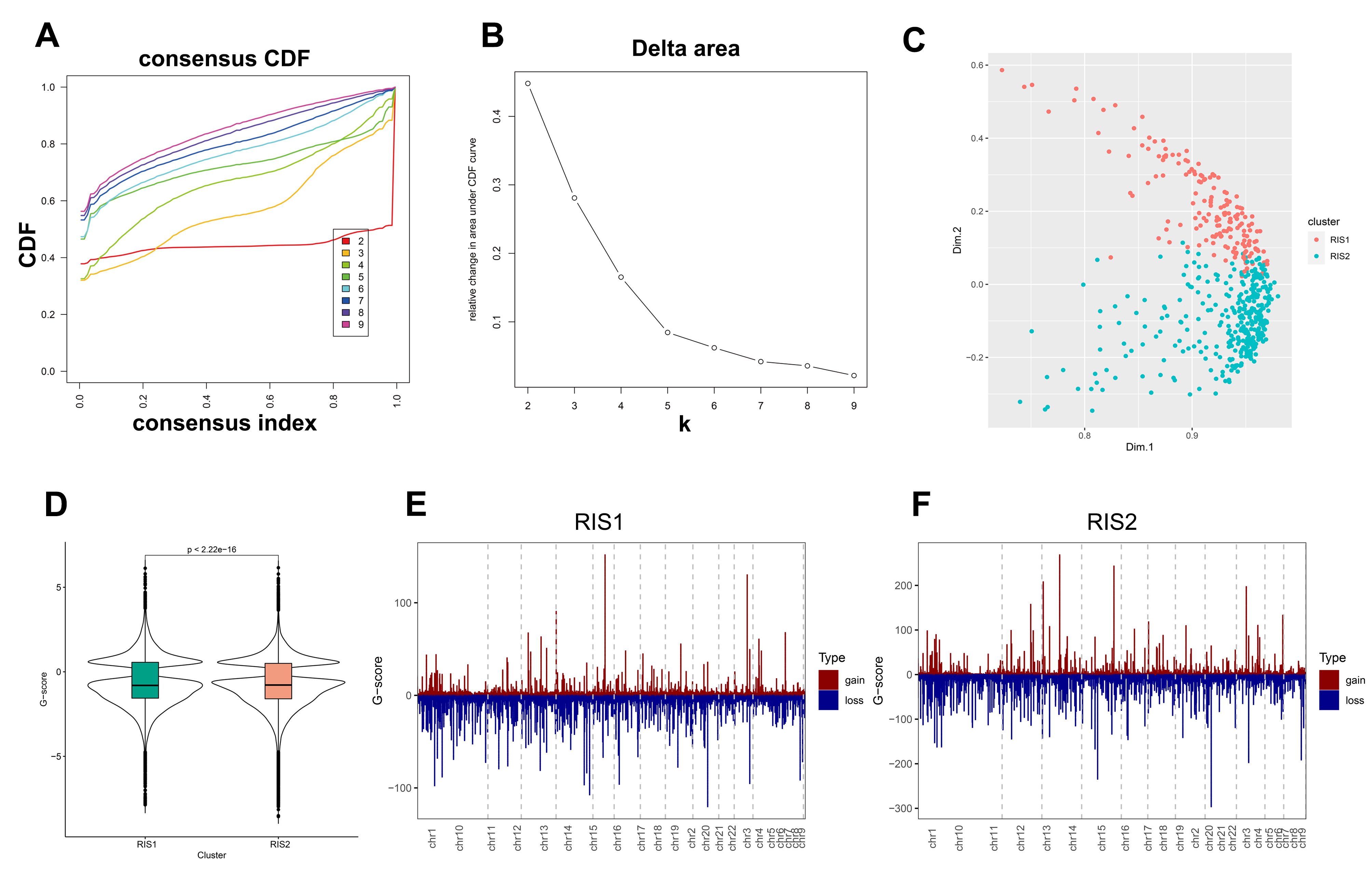

Supplement: Supplementary file 1 — Additional file 1: Figure S1. The workflow of the study. OE, overexpressed genes; APCs, antigen-presenting cells; TMB, tumor mutation burden; CNV, copy number alterations; DEGs, differentially expressed genes; RIS, renal cancer immune subtype. Figure S2. a, volcano plot; b, heatmap of overexpressed genes in normal and KIRC samples; c, overlapped genes identified through intersection; d-f, KEGG (d), Hallmark (e) and reactome (f) enrichment analysis of 572 genes after intersection of overexpressed and mutated genes. KEGG, Kyoto Encyclopedia of Genes and Genomes. Figure S3. a, cumulative distribution function curve; b, delta area of immune-related genes; c, principal component analysis; d, association of immune subtypes with G-score; e-f, Bar graph of copy number variation in RIS1 (e) and RIS2 (f). Figure S4. the differences of immune infiltration score among subtypes in immune cells. Figure S5. a-b, the differential enrichment fraction of immune cells in the above subgroups. RIS, renal cancer immune subtype; ns, not significant. * p < 0.05, ** p < 0.01, *** p < 0.001 and **** p < 0.0001. Figure S6. WGCNA module identification. a, sample clustering; b, scale-free fitting index of various soft threshold powers (β); c, the average connectivity; d, Dendrogram of all differentially expressed genes clustered based on a dissimilarity measure (1-TOM). e, number of genes in each module; f, difference distribution of feature vectors of each module in RIS1 and RIS2. RIS, renal cancer immune subtype; ns, not significant. * p < 0.05, ** p < 0.01, *** p < 0.001 and **** p < 0.0001. Figure S7. Identification of immune hub genes in KIRC. a, univariate Cox regression analysis of the 10 modules; b-d, Gene Ontology analysis of Blue (b), Yellow (c) and Green (d). Figure S8. a, risk score distribution; b, survival state distribution; c, prognosis of risk models; d, heatmap of RDX, IREB2, UBR1 and PIK3CA. Figure S9. a, heatmap of differentially expressed genes in immune subtypes; b-e, GO ( [file 12943_2021_1465_MOESM1_ESM.zip › Figure S3.tif]

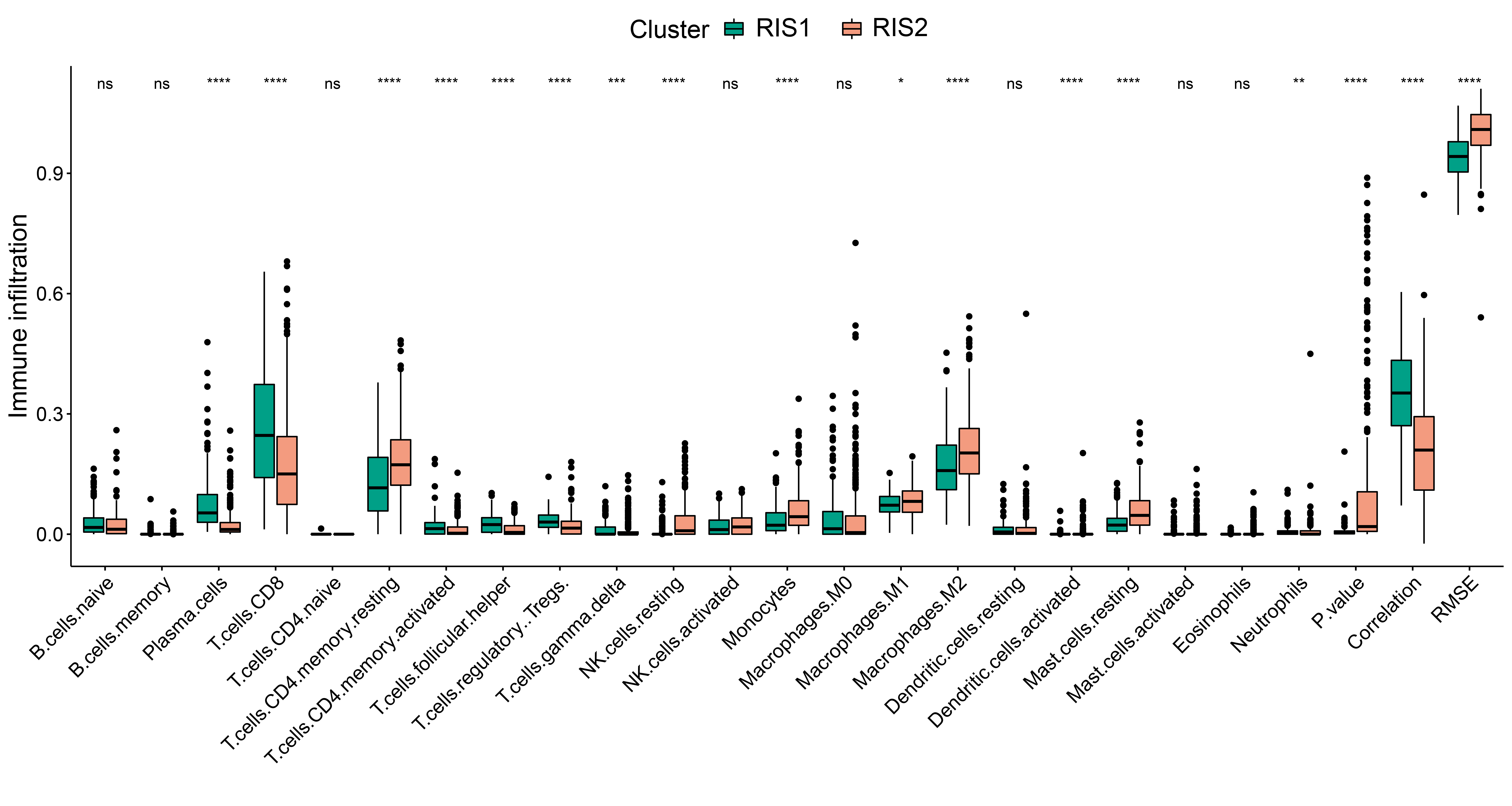

Supplement: Supplementary file 1 — Additional file 1: Figure S1. The workflow of the study. OE, overexpressed genes; APCs, antigen-presenting cells; TMB, tumor mutation burden; CNV, copy number alterations; DEGs, differentially expressed genes; RIS, renal cancer immune subtype. Figure S2. a, volcano plot; b, heatmap of overexpressed genes in normal and KIRC samples; c, overlapped genes identified through intersection; d-f, KEGG (d), Hallmark (e) and reactome (f) enrichment analysis of 572 genes after intersection of overexpressed and mutated genes. KEGG, Kyoto Encyclopedia of Genes and Genomes. Figure S3. a, cumulative distribution function curve; b, delta area of immune-related genes; c, principal component analysis; d, association of immune subtypes with G-score; e-f, Bar graph of copy number variation in RIS1 (e) and RIS2 (f). Figure S4. the differences of immune infiltration score among subtypes in immune cells. Figure S5. a-b, the differential enrichment fraction of immune cells in the above subgroups. RIS, renal cancer immune subtype; ns, not significant. * p < 0.05, ** p < 0.01, *** p < 0.001 and **** p < 0.0001. Figure S6. WGCNA module identification. a, sample clustering; b, scale-free fitting index of various soft threshold powers (β); c, the average connectivity; d, Dendrogram of all differentially expressed genes clustered based on a dissimilarity measure (1-TOM). e, number of genes in each module; f, difference distribution of feature vectors of each module in RIS1 and RIS2. RIS, renal cancer immune subtype; ns, not significant. * p < 0.05, ** p < 0.01, *** p < 0.001 and **** p < 0.0001. Figure S7. Identification of immune hub genes in KIRC. a, univariate Cox regression analysis of the 10 modules; b-d, Gene Ontology analysis of Blue (b), Yellow (c) and Green (d). Figure S8. a, risk score distribution; b, survival state distribution; c, prognosis of risk models; d, heatmap of RDX, IREB2, UBR1 and PIK3CA. Figure S9. a, heatmap of differentially expressed genes in immune subtypes; b-e, GO ( [file 12943_2021_1465_MOESM1_ESM.zip › Figure S4.tif]

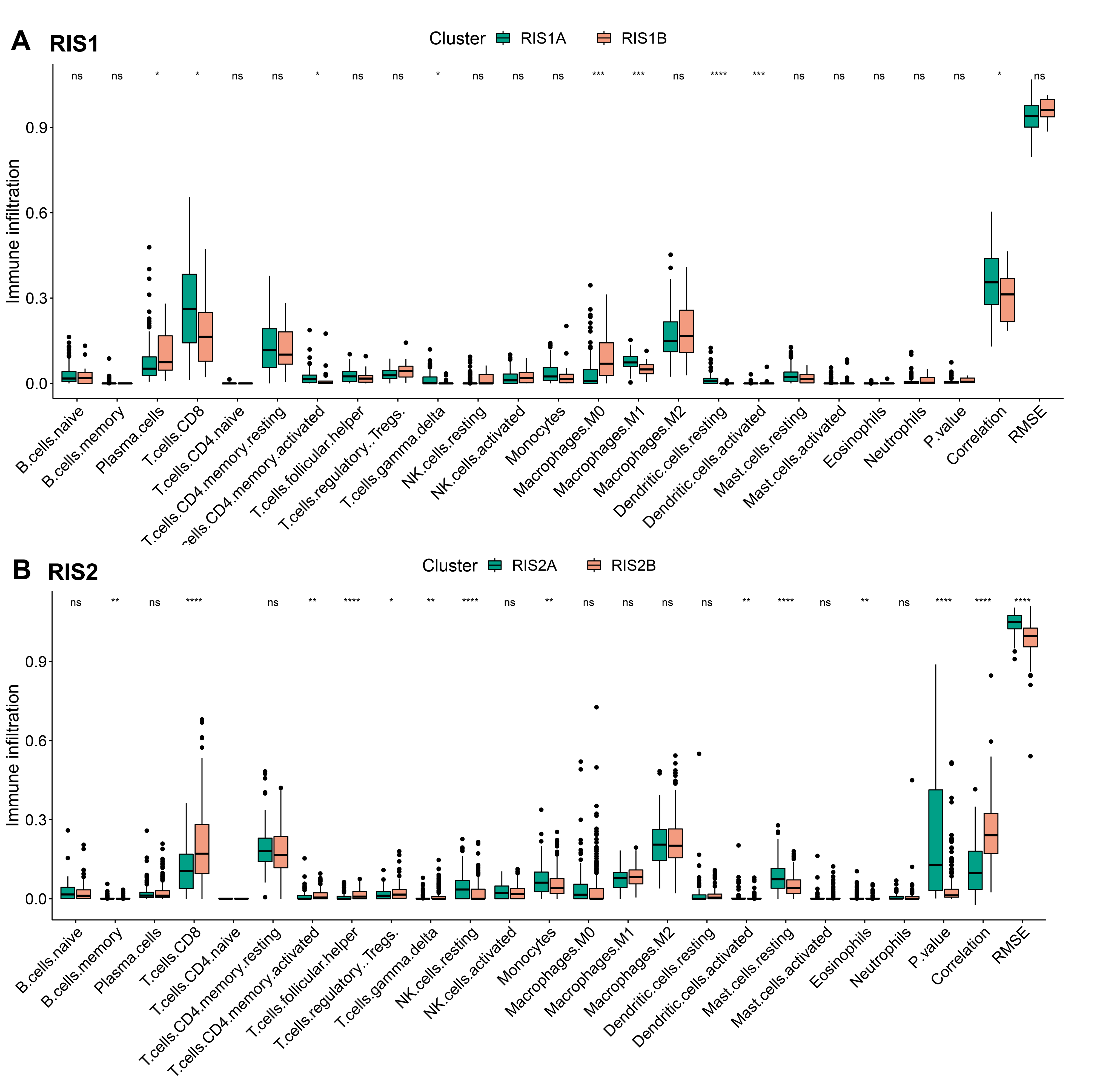

Supplement: Supplementary file 1 — Additional file 1: Figure S1. The workflow of the study. OE, overexpressed genes; APCs, antigen-presenting cells; TMB, tumor mutation burden; CNV, copy number alterations; DEGs, differentially expressed genes; RIS, renal cancer immune subtype. Figure S2. a, volcano plot; b, heatmap of overexpressed genes in normal and KIRC samples; c, overlapped genes identified through intersection; d-f, KEGG (d), Hallmark (e) and reactome (f) enrichment analysis of 572 genes after intersection of overexpressed and mutated genes. KEGG, Kyoto Encyclopedia of Genes and Genomes. Figure S3. a, cumulative distribution function curve; b, delta area of immune-related genes; c, principal component analysis; d, association of immune subtypes with G-score; e-f, Bar graph of copy number variation in RIS1 (e) and RIS2 (f). Figure S4. the differences of immune infiltration score among subtypes in immune cells. Figure S5. a-b, the differential enrichment fraction of immune cells in the above subgroups. RIS, renal cancer immune subtype; ns, not significant. * p < 0.05, ** p < 0.01, *** p < 0.001 and **** p < 0.0001. Figure S6. WGCNA module identification. a, sample clustering; b, scale-free fitting index of various soft threshold powers (β); c, the average connectivity; d, Dendrogram of all differentially expressed genes clustered based on a dissimilarity measure (1-TOM). e, number of genes in each module; f, difference distribution of feature vectors of each module in RIS1 and RIS2. RIS, renal cancer immune subtype; ns, not significant. * p < 0.05, ** p < 0.01, *** p < 0.001 and **** p < 0.0001. Figure S7. Identification of immune hub genes in KIRC. a, univariate Cox regression analysis of the 10 modules; b-d, Gene Ontology analysis of Blue (b), Yellow (c) and Green (d). Figure S8. a, risk score distribution; b, survival state distribution; c, prognosis of risk models; d, heatmap of RDX, IREB2, UBR1 and PIK3CA. Figure S9. a, heatmap of differentially expressed genes in immune subtypes; b-e, GO ( [file 12943_2021_1465_MOESM1_ESM.zip › Figure S5.tif]

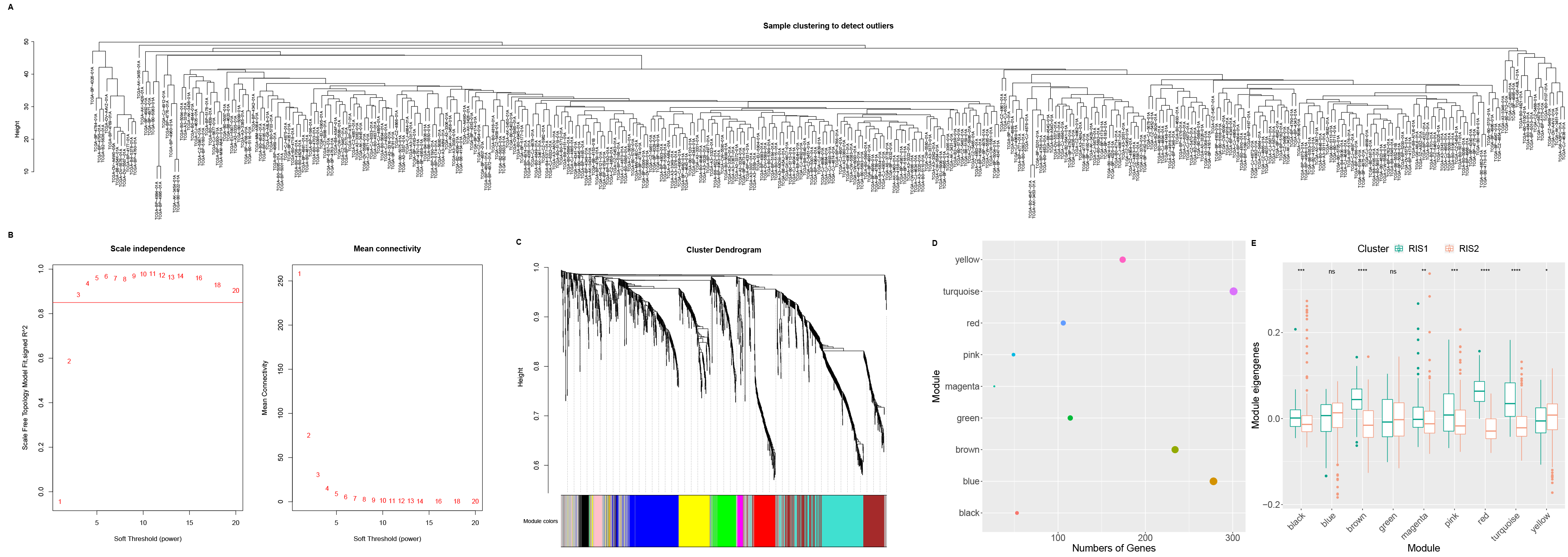

Supplement: Supplementary file 1 — Additional file 1: Figure S1. The workflow of the study. OE, overexpressed genes; APCs, antigen-presenting cells; TMB, tumor mutation burden; CNV, copy number alterations; DEGs, differentially expressed genes; RIS, renal cancer immune subtype. Figure S2. a, volcano plot; b, heatmap of overexpressed genes in normal and KIRC samples; c, overlapped genes identified through intersection; d-f, KEGG (d), Hallmark (e) and reactome (f) enrichment analysis of 572 genes after intersection of overexpressed and mutated genes. KEGG, Kyoto Encyclopedia of Genes and Genomes. Figure S3. a, cumulative distribution function curve; b, delta area of immune-related genes; c, principal component analysis; d, association of immune subtypes with G-score; e-f, Bar graph of copy number variation in RIS1 (e) and RIS2 (f). Figure S4. the differences of immune infiltration score among subtypes in immune cells. Figure S5. a-b, the differential enrichment fraction of immune cells in the above subgroups. RIS, renal cancer immune subtype; ns, not significant. * p < 0.05, ** p < 0.01, *** p < 0.001 and **** p < 0.0001. Figure S6. WGCNA module identification. a, sample clustering; b, scale-free fitting index of various soft threshold powers (β); c, the average connectivity; d, Dendrogram of all differentially expressed genes clustered based on a dissimilarity measure (1-TOM). e, number of genes in each module; f, difference distribution of feature vectors of each module in RIS1 and RIS2. RIS, renal cancer immune subtype; ns, not significant. * p < 0.05, ** p < 0.01, *** p < 0.001 and **** p < 0.0001. Figure S7. Identification of immune hub genes in KIRC. a, univariate Cox regression analysis of the 10 modules; b-d, Gene Ontology analysis of Blue (b), Yellow (c) and Green (d). Figure S8. a, risk score distribution; b, survival state distribution; c, prognosis of risk models; d, heatmap of RDX, IREB2, UBR1 and PIK3CA. Figure S9. a, heatmap of differentially expressed genes in immune subtypes; b-e, GO ( [file 12943_2021_1465_MOESM1_ESM.zip › Figure S6.tif]

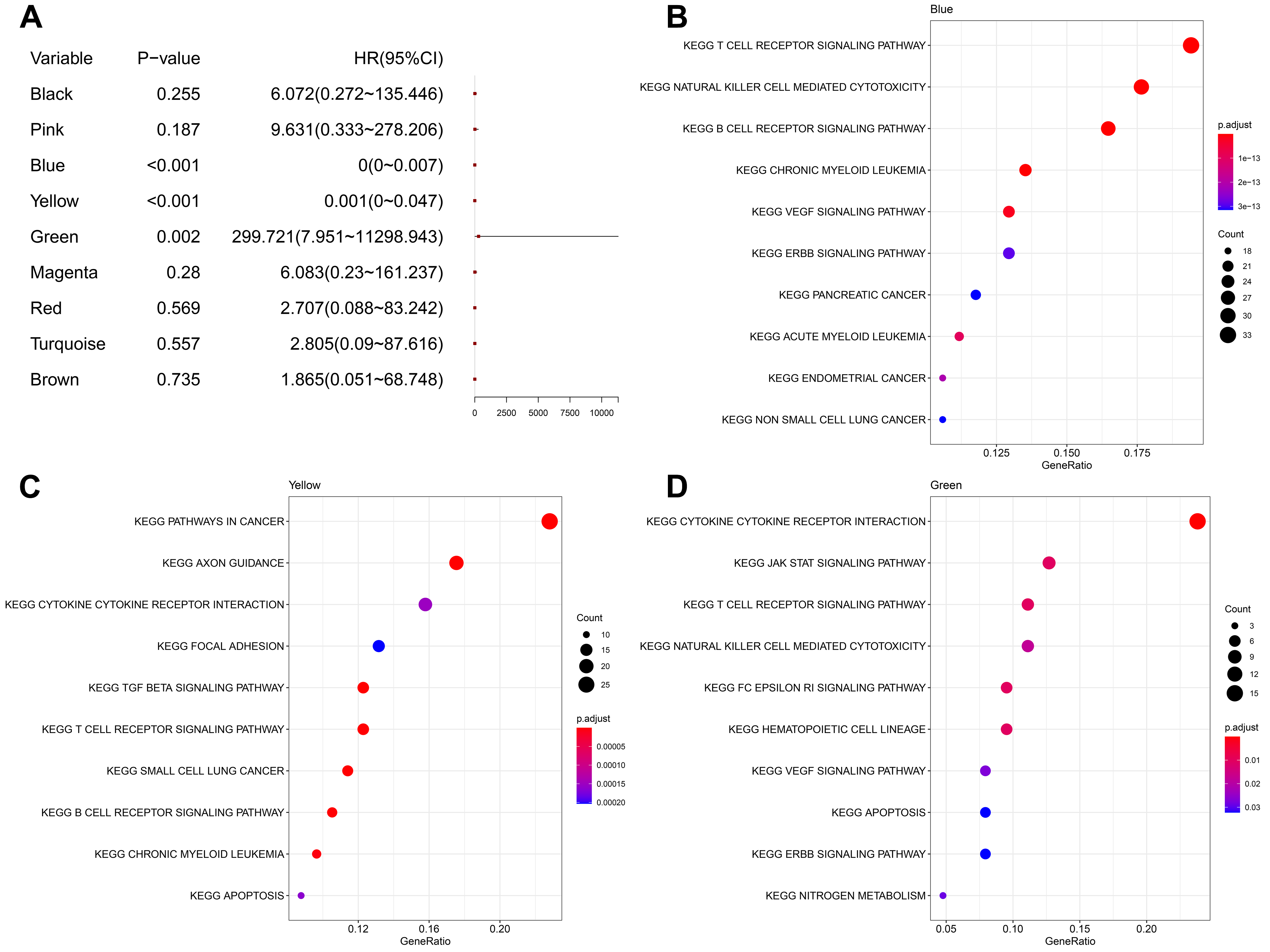

Supplement: Supplementary file 1 — Additional file 1: Figure S1. The workflow of the study. OE, overexpressed genes; APCs, antigen-presenting cells; TMB, tumor mutation burden; CNV, copy number alterations; DEGs, differentially expressed genes; RIS, renal cancer immune subtype. Figure S2. a, volcano plot; b, heatmap of overexpressed genes in normal and KIRC samples; c, overlapped genes identified through intersection; d-f, KEGG (d), Hallmark (e) and reactome (f) enrichment analysis of 572 genes after intersection of overexpressed and mutated genes. KEGG, Kyoto Encyclopedia of Genes and Genomes. Figure S3. a, cumulative distribution function curve; b, delta area of immune-related genes; c, principal component analysis; d, association of immune subtypes with G-score; e-f, Bar graph of copy number variation in RIS1 (e) and RIS2 (f). Figure S4. the differences of immune infiltration score among subtypes in immune cells. Figure S5. a-b, the differential enrichment fraction of immune cells in the above subgroups. RIS, renal cancer immune subtype; ns, not significant. * p < 0.05, ** p < 0.01, *** p < 0.001 and **** p < 0.0001. Figure S6. WGCNA module identification. a, sample clustering; b, scale-free fitting index of various soft threshold powers (β); c, the average connectivity; d, Dendrogram of all differentially expressed genes clustered based on a dissimilarity measure (1-TOM). e, number of genes in each module; f, difference distribution of feature vectors of each module in RIS1 and RIS2. RIS, renal cancer immune subtype; ns, not significant. * p < 0.05, ** p < 0.01, *** p < 0.001 and **** p < 0.0001. Figure S7. Identification of immune hub genes in KIRC. a, univariate Cox regression analysis of the 10 modules; b-d, Gene Ontology analysis of Blue (b), Yellow (c) and Green (d). Figure S8. a, risk score distribution; b, survival state distribution; c, prognosis of risk models; d, heatmap of RDX, IREB2, UBR1 and PIK3CA. Figure S9. a, heatmap of differentially expressed genes in immune subtypes; b-e, GO ( [file 12943_2021_1465_MOESM1_ESM.zip › Figure S7.tif]

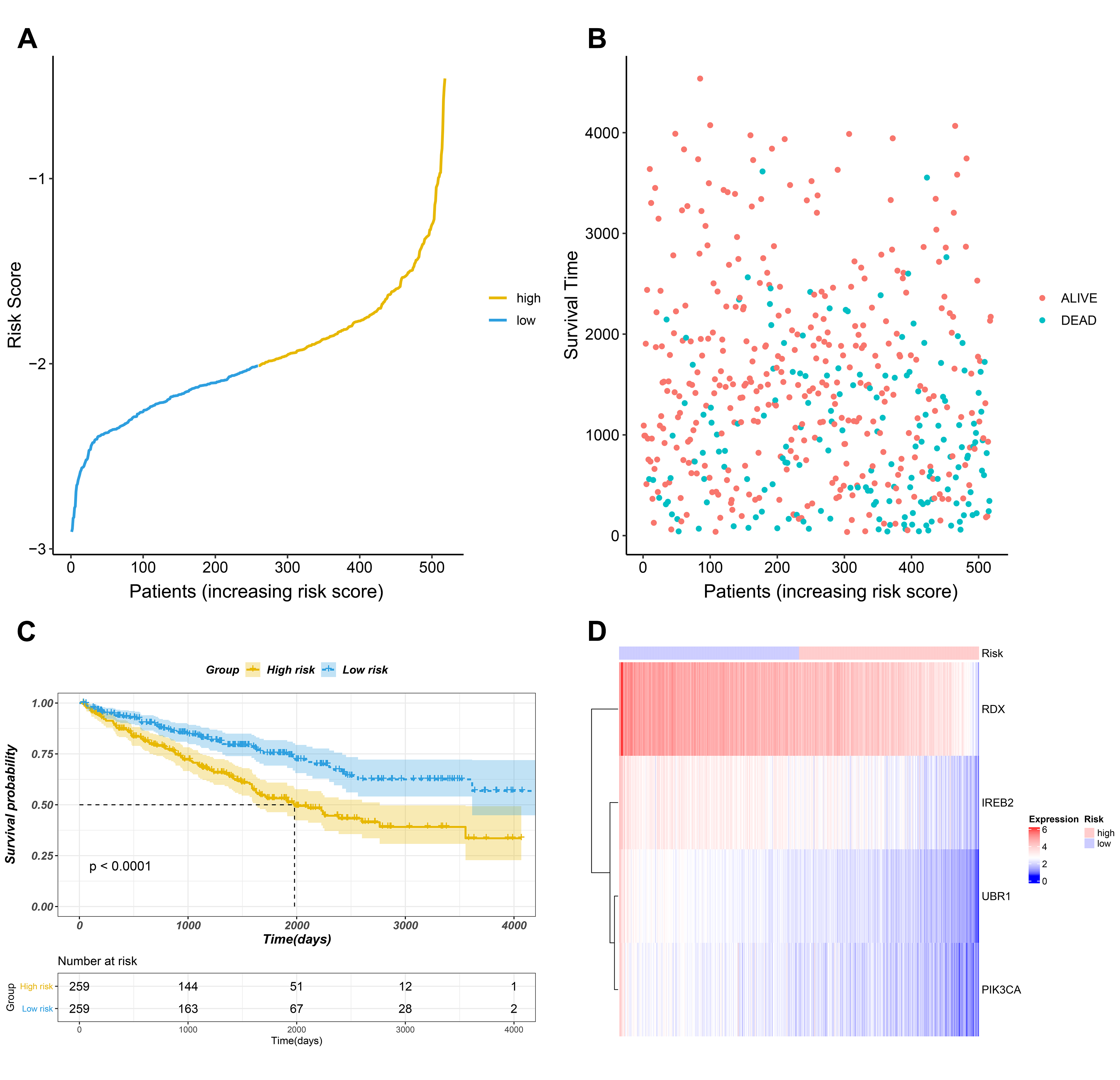

Supplement: Supplementary file 1 — Additional file 1: Figure S1. The workflow of the study. OE, overexpressed genes; APCs, antigen-presenting cells; TMB, tumor mutation burden; CNV, copy number alterations; DEGs, differentially expressed genes; RIS, renal cancer immune subtype. Figure S2. a, volcano plot; b, heatmap of overexpressed genes in normal and KIRC samples; c, overlapped genes identified through intersection; d-f, KEGG (d), Hallmark (e) and reactome (f) enrichment analysis of 572 genes after intersection of overexpressed and mutated genes. KEGG, Kyoto Encyclopedia of Genes and Genomes. Figure S3. a, cumulative distribution function curve; b, delta area of immune-related genes; c, principal component analysis; d, association of immune subtypes with G-score; e-f, Bar graph of copy number variation in RIS1 (e) and RIS2 (f). Figure S4. the differences of immune infiltration score among subtypes in immune cells. Figure S5. a-b, the differential enrichment fraction of immune cells in the above subgroups. RIS, renal cancer immune subtype; ns, not significant. * p < 0.05, ** p < 0.01, *** p < 0.001 and **** p < 0.0001. Figure S6. WGCNA module identification. a, sample clustering; b, scale-free fitting index of various soft threshold powers (β); c, the average connectivity; d, Dendrogram of all differentially expressed genes clustered based on a dissimilarity measure (1-TOM). e, number of genes in each module; f, difference distribution of feature vectors of each module in RIS1 and RIS2. RIS, renal cancer immune subtype; ns, not significant. * p < 0.05, ** p < 0.01, *** p < 0.001 and **** p < 0.0001. Figure S7. Identification of immune hub genes in KIRC. a, univariate Cox regression analysis of the 10 modules; b-d, Gene Ontology analysis of Blue (b), Yellow (c) and Green (d). Figure S8. a, risk score distribution; b, survival state distribution; c, prognosis of risk models; d, heatmap of RDX, IREB2, UBR1 and PIK3CA. Figure S9. a, heatmap of differentially expressed genes in immune subtypes; b-e, GO ( [file 12943_2021_1465_MOESM1_ESM.zip › Figure S8.tif]

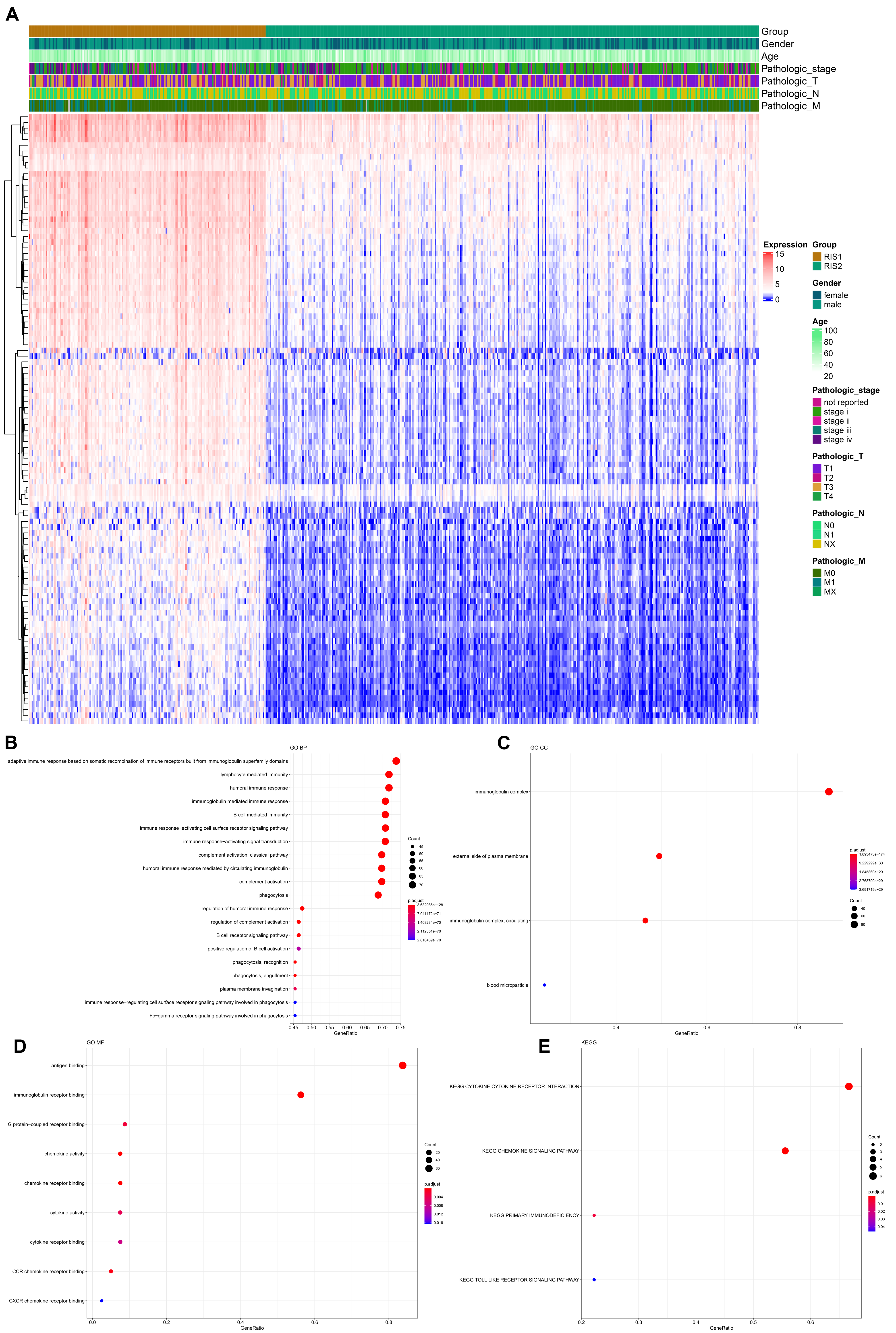

Supplement: Supplementary file 1 — Additional file 1: Figure S1. The workflow of the study. OE, overexpressed genes; APCs, antigen-presenting cells; TMB, tumor mutation burden; CNV, copy number alterations; DEGs, differentially expressed genes; RIS, renal cancer immune subtype. Figure S2. a, volcano plot; b, heatmap of overexpressed genes in normal and KIRC samples; c, overlapped genes identified through intersection; d-f, KEGG (d), Hallmark (e) and reactome (f) enrichment analysis of 572 genes after intersection of overexpressed and mutated genes. KEGG, Kyoto Encyclopedia of Genes and Genomes. Figure S3. a, cumulative distribution function curve; b, delta area of immune-related genes; c, principal component analysis; d, association of immune subtypes with G-score; e-f, Bar graph of copy number variation in RIS1 (e) and RIS2 (f). Figure S4. the differences of immune infiltration score among subtypes in immune cells. Figure S5. a-b, the differential enrichment fraction of immune cells in the above subgroups. RIS, renal cancer immune subtype; ns, not significant. * p < 0.05, ** p < 0.01, *** p < 0.001 and **** p < 0.0001. Figure S6. WGCNA module identification. a, sample clustering; b, scale-free fitting index of various soft threshold powers (β); c, the average connectivity; d, Dendrogram of all differentially expressed genes clustered based on a dissimilarity measure (1-TOM). e, number of genes in each module; f, difference distribution of feature vectors of each module in RIS1 and RIS2. RIS, renal cancer immune subtype; ns, not significant. * p < 0.05, ** p < 0.01, *** p < 0.001 and **** p < 0.0001. Figure S7. Identification of immune hub genes in KIRC. a, univariate Cox regression analysis of the 10 modules; b-d, Gene Ontology analysis of Blue (b), Yellow (c) and Green (d). Figure S8. a, risk score distribution; b, survival state distribution; c, prognosis of risk models; d, heatmap of RDX, IREB2, UBR1 and PIK3CA. Figure S9. a, heatmap of differentially expressed genes in immune subtypes; b-e, GO ( [file 12943_2021_1465_MOESM1_ESM.zip › Figure S9.tif]
